# The histamine H_3_ receptor inverse agonist pitolisant reduces body weight in obese mice

**DOI:** 10.1007/s00210-018-1516-2

**Published:** 2018-05-25

**Authors:** Magdalena Kotańska, Kamil J. Kuder, Katarzyna Szczepańska, Jacek Sapa, Katarzyna Kieć-Kononowicz

**Affiliations:** 1 Department of Pharmacodynamics, Jagiellonian University Medical College, 9 Medyczna Street, 30-688 Krakow, Poland; 2 Department of Technology and Biotechnology of Drugs, Faculty of Pharmacy, Jagiellonian University Medical College, Krakow, Poland; 3 Department of Pharmacological Screening, Jagiellonian University Medical College, 9 Medyczna Street, 30-688 Krakow, Poland

**Keywords:** Pitolisant, H_3_ histamine ligand, Obesity, Glucose tolerance, Metabolic disturbance

## Abstract

The pharmacological profile of pitolisant, a histamine H_3_ receptor antagonist/inverse agonist, indicates that this compound might reduce body weight and metabolic disturbances. Therefore, we studied the influence of pitolisant on body weight, water and sucrose intake as well as metabolic disturbances in the high-fat and high-sugar diet-induced obesity model in mice. To induce obesity, male CD-1 mice were fed a high-fat diet consisting of 40% fat blend for 14 weeks, water and 30% sucrose solution available ad libitum. Glucose tolerance test was performed at the beginning of week 15. Insulin tolerance was tested the day after. At the end of study, plasma levels of triglycerides and cholesterol were determined. Pitolisant at dose of 10 mg/kg bw (ip) was administrated during 14 days, starting from the beginning of week 13. Metformin at dose of 100 mg/kg bw (ip) was used as reference drug. Mice fed with high-fat diet and sucrose solution showed more weight gain throughout the 12-week period of inducing obesity. Animals fed with high-fat diet and treated with pitolisant (for the next 14 days) showed significantly less weight gain than mice from the control group consuming a high-fat feed. In the group treated with pitolisant, glucose levels were significantly lower than glucose levels of control obese mice after glucose load. The plasma triglyceride levels in pitolisant-treated mice were significantly lower compared with those in control obese group. In conclusion, pitolisant has a favorable influence of body weight and improves glucose tolerance and the lipid profile in obese mice.

## Introduction

Histamine H_3_ receptors serve as presynaptic inhibitory autoreceptors in the central nervous system; inverse agonists and antagonists of these receptors increase the synthesis and release of histamine (Walter and Stark 2012). Histamine itself might regulate food intake and metabolic disturbances by influencing the histamine H_1_, H_2_ and H_3_ receptors (Provensi et al. 2016). Moreover, histamine regulates the release and interaction of other neurotransmitters such as dopamine, acetylcholine, serotonin, norepinephrine, γ-aminobutyric acid, glutamate and substance P by affecting histamine H_3_ heteroreceptors (Feuerstein 2008). Through this pathway, it controls indirectly, e.g. food intake and motor activity. Histamine also affects the peripheral metabolism by increasing white adipose tissue lipolysis (Passani et al. 2011).

Histamine H_1_ and H_2_ receptor signalling pathways play important roles in glucose and lipid metabolism, which seems to be mediated through both central and peripheral pathways. Histamine H_1_ receptor signalling is involved in central nervous system and pancreatic tissue to regulate glucose metabolism whereas histamine H_2_ receptors regulate lipid and glucose metabolisms in the liver and skeletal muscle via the adiponectin system (Wang et al. 2010). In addition, compounds that block histamine H_3_ receptor activity are capable of reducing the level of triglycerides in plasma (Malmlöf et al. 2006). In peripheral tissues, H_3_ histamine receptors are expressed in neuroendocrine organs and regulate their functions, e.g. in pancreatic β cells in mouse and human and play an important role in insulin secretion (Nakamura et al. 2014).

Previous studies indicated that H_3_ histamine receptor ligands might exhibit potential anti-obesity activity (Provensi et al. 2016). The H_3_ histamine receptor antagonists (i.e. NNC 38-1049, NNC 38-1202, JNJ-5207852, GT-2394, A-423579; A-631972, A-331440) supress food intake and cause profound weight loss in various obesity rodent models (Barbier et al. 2004; Yates et al. 2002; Hancock and Brune 2005; Malmlöf et al. 2005, 2006). Betahistine, a partial inverse H_3_ histamine receptor agonist, induced significant weight loss with minimal adverse events in women under 50 years of age (Barak et al. 2008) and in animals with obesity after olanzapine treatment (Lian et al. 2014).

**Fig. 1:**
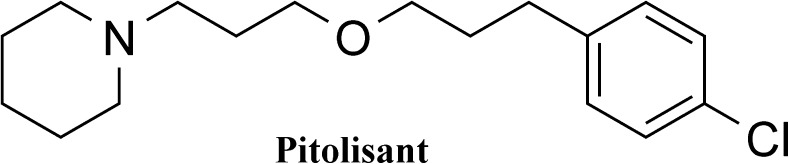
Chemical structure of pitolisant

Pitolisant (Fig. 1) is an antagonist/inverse agonist at the H_3_ histamine receptor (EC_50_ (human H_3_ receptor) = 1.5 nM) (EMA 2015) and might reduce body weight and metabolic disturbances and be beneficial in the treatment of obesity. Figure 1 shows the structure. Therefore, in the present study, we aimed to evaluate the influence of pitolisant on body weight, water and sucrose intake and metabolic disturbances in high-fat and high-sugar diet-induced obesity model in mice.

## Materials and methods

### Animals

Adult male Albino Swiss mice, CD-1, weighing 20–22 g were used in the study. Animals were kept in environmentally controlled rooms, in standard cages lit by an artificial light for 12 h each day. Animals had free access to food and water, except for the time of the acute experiment. The randomly established experimental groups consisted of six mice. All animal care and experimental procedures were carried out in accordance with European Union and Polish legislation acts concerning animal experimentation and were approved by the Local Ethics Committee at the Jagiellonian University in Cracow, Poland (Permission No: 135/2013).

### Experimental methods

#### Metabolic disturbance induced with a high-fat/sucrose diet and influence of pitolisant on body weight

**Fig. 2:**
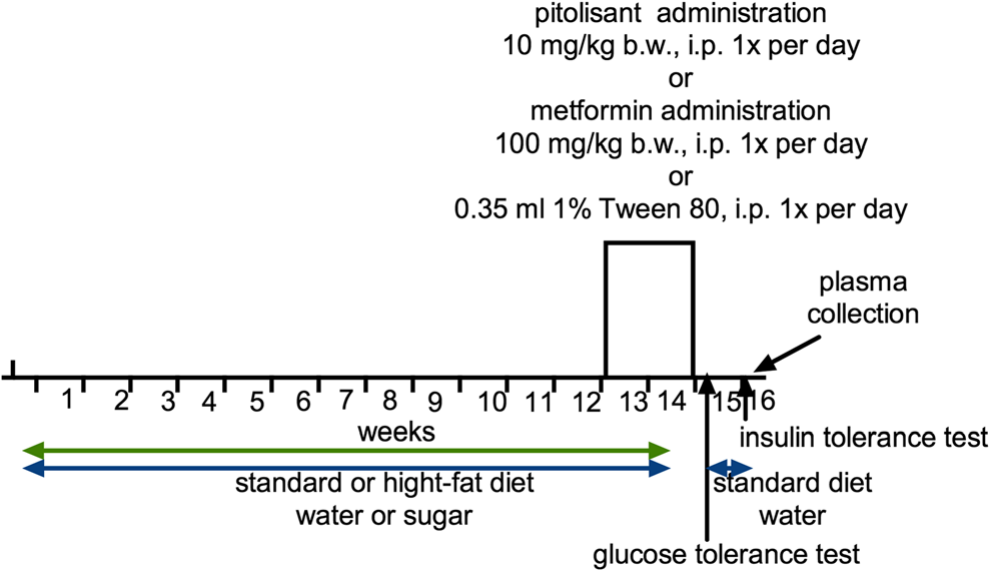
Scheme of the experiment

Male CD-1 mice were fed on high-fat diet consisting of 40% fat blend (Labofeed B with 40% lard, Morawski Feed Manufacturer, Poland) for 14 weeks, water and 30% sucrose available ad libitum. Control mice were fed on a standard diet (Labofeed B, Morawski Feed Manufacturer, Poland) and drank water only. After 12 weeks, mice with obesity induced via their diet were randomly divided into three equal groups that had the same mean body weight and were treated intraperitoneally with test compounds at the following doses: pitolisant 10 mg/kg bw/day (Dudek et al. 2016; Uguen et al. 2013; Zhang et al. 2012) or metformin 100 mg/kg bw/day (Al-Barazanji et al. 2015; Tahara et al. 2011); control group: vehicle =1% Tween 80, 0.35 ml/kg (high-fat/sugar diet + vehicle = obesity control group) once daily in the morning, between 9:00 and 10:00 AM for 14 days. Control mice (control without obesity) were maintained on a standard diet, with intraperitoneal administration of vehicle = 1% Tween 80, 0.35 ml/kg (standard diet + vehicle = control group). Water and sucrose were measured daily, immediately prior to administration of drugs. Animals always had free access to feed, water and sucrose. Figure 2 shows a scheme that depicts the entire time course of the experiment.

High-fat feed composition (932 g of dry mass): protein = 193 g, fat (lard) = 408 g, fibre = 28.1 g, crude ash = 43.6 g, calcium = 9.43 g, phosphorus = 5.99 g, sodium = 1.76 g, sugar = 76 g, magnesium = 1.72 g, potassium = 7.62 g, manganese = 48.7 mg, iodine = 0.216 mg, copper = 10.8 mg, iron = 125 mg, zinc = 61.3 mg, cobalt = 0.253 mg, selenium = 0.304 mg, vitamin A = 15,000 units, vitamin D_3_ = 1000 units, vitamin E = 95.3 mg, vitamin K_3_ = 3.0 mg, vitamin B_1_ = 8.06 mg, vitamin B_2_ = 6.47 mg, vitamin B_6_ = 10.3 mg, vitamin B_12_ = 0.051 mg, folic acid = 2.05 mg, nicotinic acid = 73.8 mg, pantothenic acid = 19.4 mg and choline = 1578 mg. The high-fat diet contained 550 kcal and the standard diet 280 kcal/100 g.

#### Glucose tolerance test

The test was performed at the beginning of week 15. After 14th administration of the test compound, food and sucrose were discontinued for 20 h and after this time, glucose tolerance was tested. Glucose (1 g/kg bw) was administrated intraperitoneally. Blood samples were taken at time points 0 (before glucose administration) and 30, 60 and 120 min after administration from tail vein. Glucose level was measured with glucometer (ContourTS, Bayer, Germany, test stripes: ContourTS, Ascensia Diabetes care Poland Sp. z o.o., Poland, REF: 84239666). The area under the curve (AUC) was calculated using the trapezoid rule.

#### Insulin tolerance test

Insulin tolerance was tested the next day after the glucose tolerance test. After the test, mice had free access to standard food and water. Also, 3 h before insulin tolerance test, the food was discontinued. Insulin (0.5 IU/kg bw) was injected intraperitoneally and blood samples were taken at time points: 0, 15 and 30 min from tail vein and glucose level was measured with glucometer (ContourTS, Bayer, Germany, test stripes: ContourTS, Ascensia Diabetes care Poland Sp. z o.o., Poland, REF:84239666). The AUC was calculated using the trapezoid rule.

#### Biochemical analysis

The blood was collected after decapitation and then centrifuged at 1200 rpm (15 min) to obtain the plasma. To determine cholesterol and triglyceride levels in the plasma, standard enzymatic spectrophotometric tests (Biomaxima S.A. Lublin, Poland, catalogue number: 1-023-0400 or 1-053-0400) were used. The substrate was decomposed with enzymes appropriate for the relevant product and converted to a coloured compound. Absorbance was measured at a wavelength of 500 nm.

##### Cholesterol

Cholesterol esterase hydrolyses cholesterol esters to free cholesterol and fatty acids. In the presence of cholesterol oxidase, free cholesterol is oxidized to cholest-4-en-3-one and hydrogen peroxide. Hydrogen peroxide reacts with phenol and 4-aminoantipyrine to form a coloured complex. The intensity of the colour is directly proportional to the cholesterol concentration.

##### Triglyceride

Lipase hydrolyzes triglycerides to glycerol and fatty acids. Glycerol in the presence of glycerol kinase and ATP is phosphorylated to 3-P-glycerol. 3-P-glycerol oxidase catalyzes the H_2_O_2_ formation. Hydrogen peroxide reacts with 4-chlorophenol and 4-aminoantipyrine to form a coloured complex. The intensity of the colour is directly proportional to the concentration of triglycerides.

### Statistical analysis

The results obtained were analysed using a one-way variance analysis (ANOVA), followed by a Dunnett post hoc test, with the significance level set at 0.05 (triglyceride and cholesterol levels) or a two-way variance analysis (ANOVA), followed by a Bonferroni post hoc test (changes of body weight), with the significance level set at 0.05 or by a Multiple *t* test under the assumption, that all rows were sampled from populations with the same scatter (glucose tolerance test, insulin tolerance test). The results were expressed as the means ± standard error of the mean (SEM). Graph Pad Prism 6.0 was used for data analysis.

### Drugs, chemical reagents and other materials

Metformin was purchased from Teva Pharmaceuticals (Poland). Pitolisant was synthesized in the Department of Technology and Biotechnology of Drugs, Faculty of Pharmacy, Jagiellonian University Medical College, Krakow, Poland, according to procedure described by Meier et al. (2001). Identity and purity of final product were assessed by NMR and LC-MS techniques.

Pitolisant (10 mg/kg bw) and metformin (100 mg/kg bw) were suspended in 1% Tween 80. The compounds or vehicle were administered intraperitoneally (ip) once daily. The volume of the vehicle or the drug solutions was 10 ml/kg.

## Results

### Influence of pitolisant on body weight

**Fig. 3:**
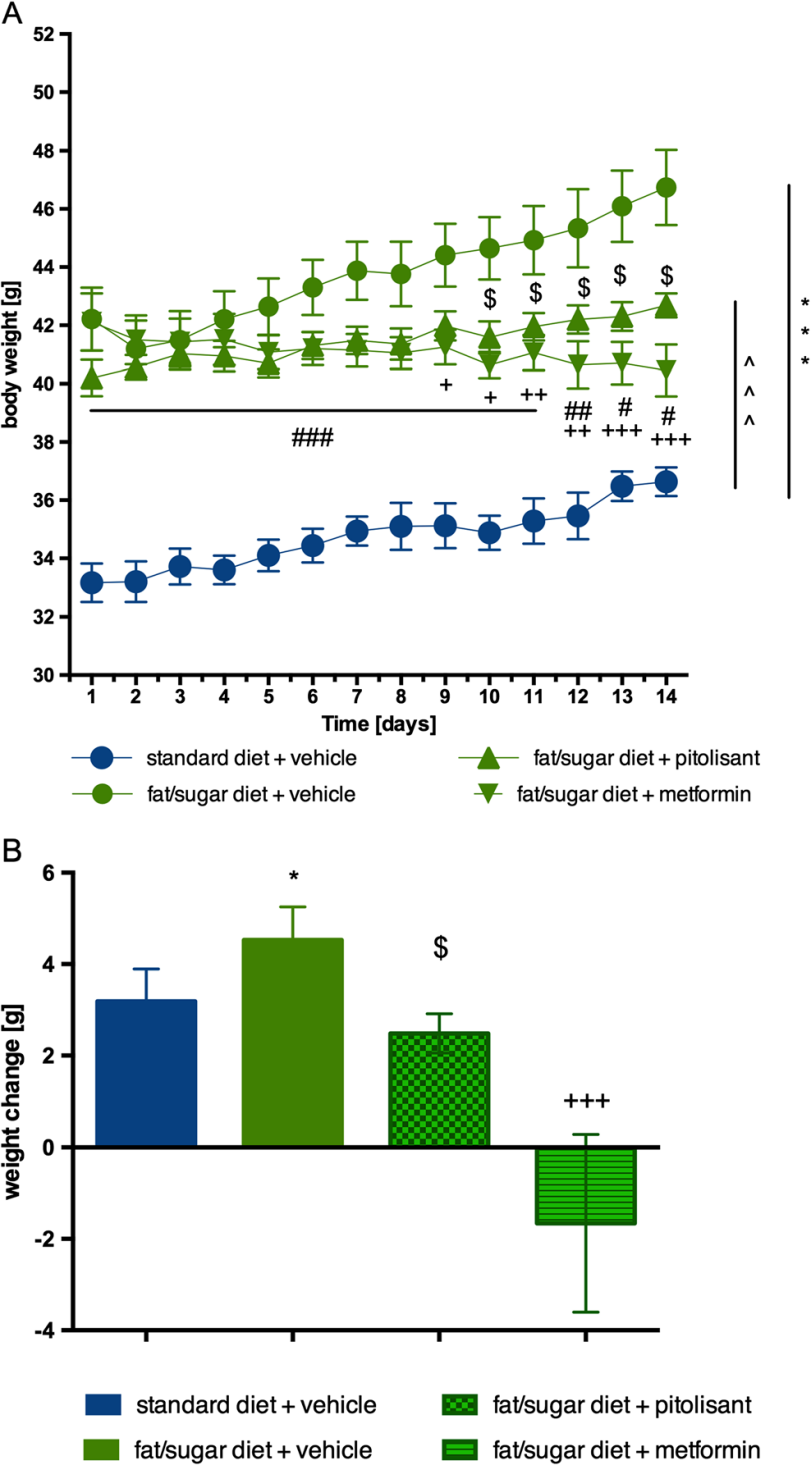
Effect of administration of pitolisant on body weight. **a** Body weight throughout the administration. **b** Sum of weight changes. Results are means ± SEM, *n* = 6. Multiple comparisons were performed by two-way ANOVA, Bonferroni post-hoc (**a**) or one-way ANOVA Tukey post hoc (**b**). *p < 0.05, ***p < 0.001 significant between control mice fed with fat diet vs. control mice fed with standard diet; ^^^*p* < 0.001 significant between control mice fed with standard diet vs. mice treated with pitolisant; $*p* < 0.05 significant between control mice fed with fat diet vs. mice treated with pitolisant; +*p* < 0.05, ++*p* < 0.01 and +++*p* < 0.001 significant between control mice fed with fat diet vs. mice treated with metformin; #*p* < 0.05, ##*p* < 0.01 and ###*p* < 0.001 significant between control mice fed with standard diet vs. mice treated with metformin

Mice fed with high-fat diet showed more weight gain throughout the 12-week period of inducing obesity. Animals fed with high-fat diet and treated with pitolisant (next 14 days) showed significantly less weight gain than mice from the control group consuming a high-fat diet. From the 10th day of the pitolisant administration, a statistically significant difference in body weight between the groups was observed. Metformin, which served as a positive control, reduced body weight from the ninth day of administration onward. Results are shown in Fig. 3.

### Influence of pitolisant on water and sucrose intake

**Fig. 4:**
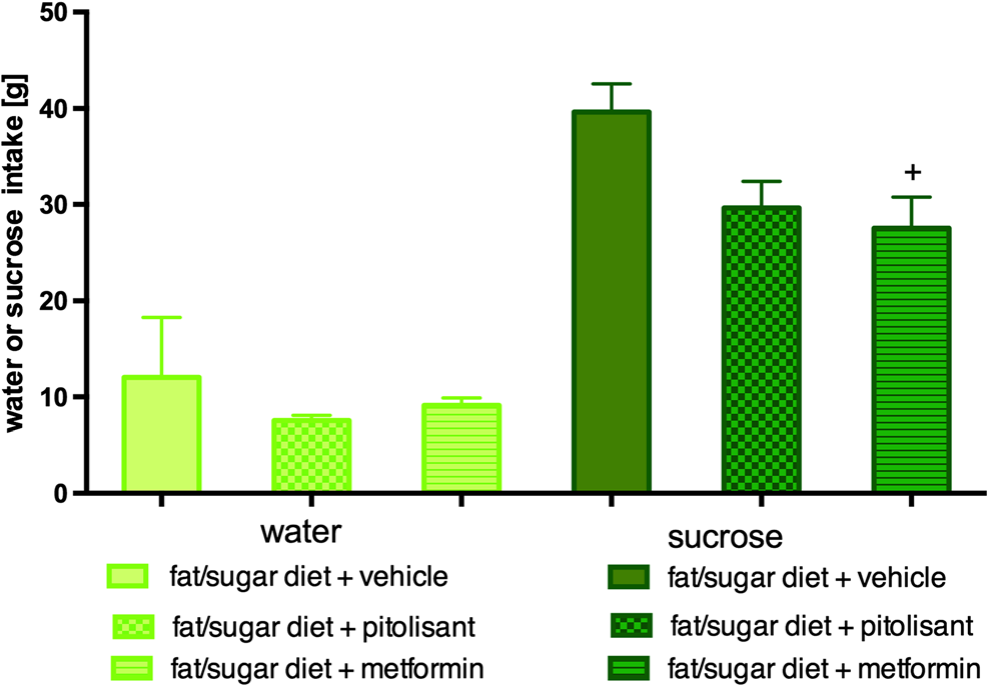
Effect of administration of pitolisant on sucrose intake and water in CD-1 mice. Average of water intake and sucrose. Results are means ± SEM, *n* = 6. Comparisons were performed by one-way ANOVA Dunnett post hoc; +*p* < 0.05 significant between control mice fed with fat diet vs. mice treated with metformin

There were no significant differences in water intake by animals between groups fed with high-fat diet. There was no significant difference in sucrose intake by mice treated with pitolisant vs. control obese mice fed with high-fat diet. Significantly less sucrose solution was drunk by animals fed on high-fat diet and treated with metformin vs. control obese mice fed with high-fat diet. Results are shown in Fig. 4.

### Glucose tolerance and insulin sensitivity after pitolisant treatment of obese mice

**Fig. 5:**
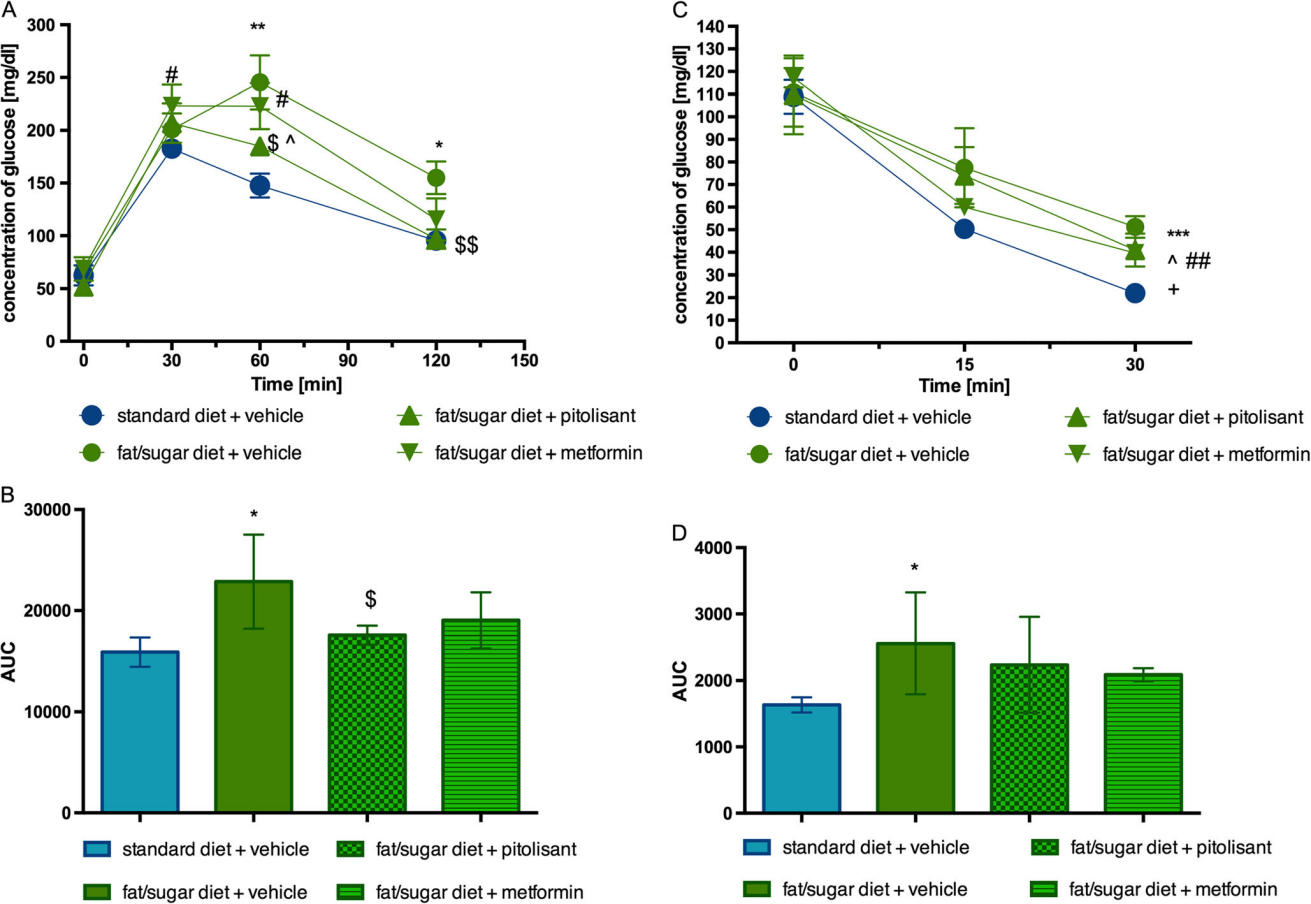
Glucose tolerance and insulin sensitivity. **a** Intraperitoneal glucose tolerance test (IPGTT). **b** Area under the curve of IPGTT. **c** Insulin tolerance test (ITT). **d** Area under the curve of the ITT. Results are means ± SEM, *n* = 6. Comparisons were performed by two-way ANOVA, Bonferroni post hoc. *p < 0.05, **p < 0.01 and ***p < 0.001 significant between control mice fed with fat diet vs. control mice fed standard diet; $*p* < 0.05 and $$*p* < 0.01 significant between control mice fed with fat diet vs. mice treated with pitolisant; ^*p* < 0.05 significant between control mice fed with standard diet vs. mice treated with pitolisant; #*p* < 0.05 and ##*p* < 0.01 significant between control mice fed with standard diet vs. mice treated with metformin; +*p* < 0.05 significant between control mice fed with fat diet vs. mice treated with metformin

Blood glucose levels of control obese mice fed with high-fat diet at 60 and 120 min were significantly higher after the glucose load compared with control mice (fed with standard feed). In group treated with pitolisant at dose of 10 mg/kg bw for 14 days, glucose levels were significantly reduced after glucose load in obese mice. The blood glucose levels at 60 and 120 min were significantly lower vs. glucose levels of control obese mice and there were no significant differences among pitolisant treated group and control group (fed with standard feed) at 30 and 120 min. Metformin behaved like pitolisant but the effects of the former drug did not reach a significant level (Fig. 5a, b). As shown in Fig. 5b, the AUC was also significantly decreased by pitolisant treatment compared with obese control group.

In the insulin test (30 min), metformin slightly but significantly decreased blood glucose of obese mice whereas pitolisant only tended to do so (Fig. 5c). If the AUCs are considered neither drug showed a significant effect (Fig. 5d).

### Influence of pitolisant on triglyceride and cholesterol levels

**Fig. 6:**
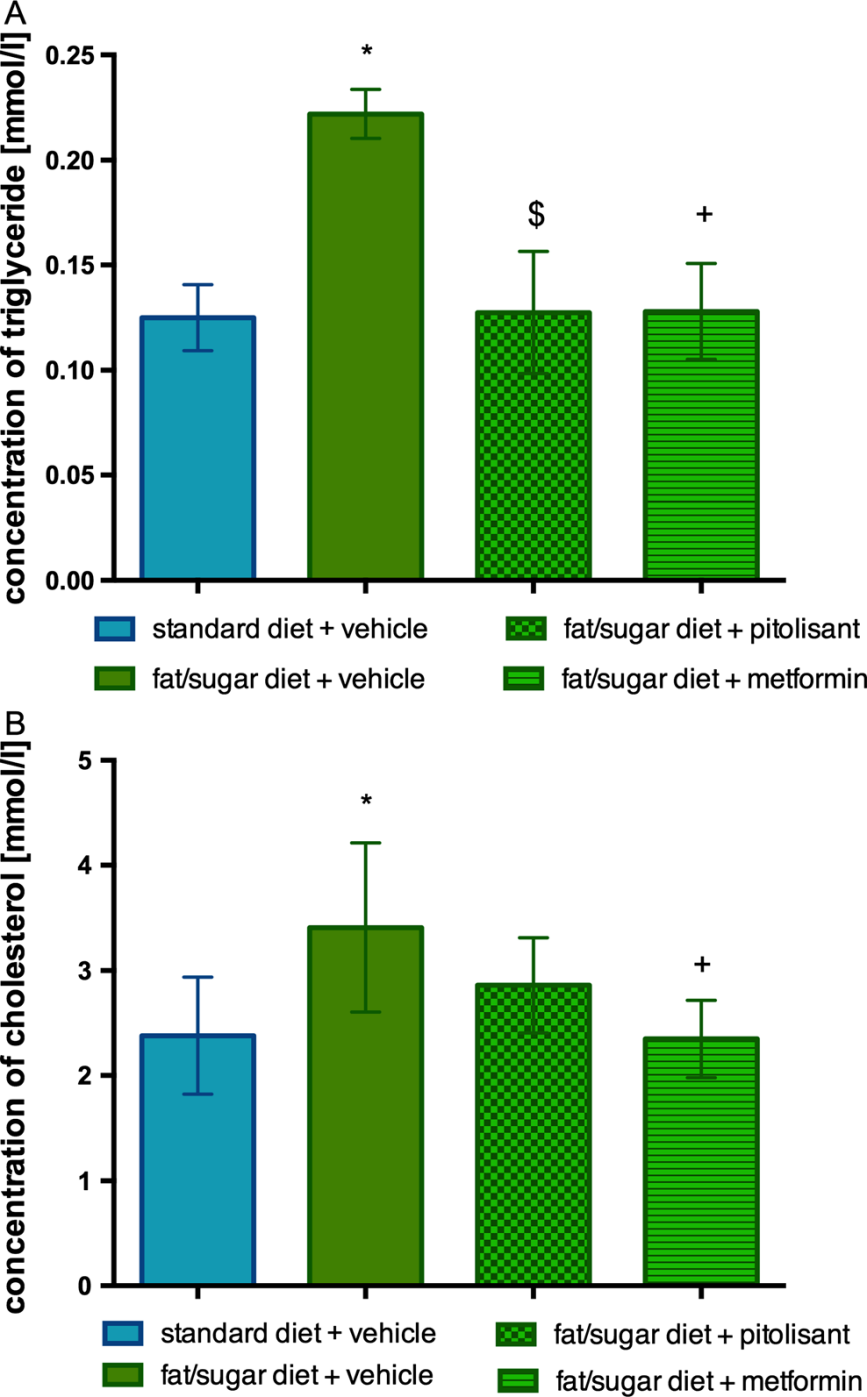
Effects of administration of pitolisant on plasma triglyceride level in CD-1 mice (**a**) and on plasma choleresterol level in CD-1 mice (**b**). Results are means ± SEM, *n* = 6. Comparisons were performed by one-way ANOVA Dunnett post hoc. *p < 0.05 significant between control mice fed with fat diet vs. control mice fed standard diet; $*p* < 0.05, significant between control mice fed with fat diet vs. mice treated with pitolisant; +*p* < 0.05 significant between control mice fed with fat diet vs. mice treated with metformin

Hyperlipidemia is a characteristic feature of fat/sugar-induced obesity in mice. Cholesterol and triglyceride levels were elevated in obese individuals. As shown in Fig. 6a, the plasma triglyceride levels in pitolisant- and metformin-treated mice were significantly lower compared to the level in control obese group. There was no significant difference vs. control group fed with standard feed. The plasma cholesterol level of the control obese group tended to be decreased by pitolisant; metformin led to a significant reduction (Fig. 6b).

## Discussion

Although a number of studies with histaminergic ligands in order to search for a safe and effective drug that might reduce body weight at obese individuals were conducted (Provensi et al. 2016), up to date, there was no literature data on pitolisant, the only histamine H_3_ receptor inverse agonist that reached the marked. Therefore, in this study, we performed screening tests that evaluate the impact of pitolisant on body weight and metabolic disorders of obese mice.

Our research has shown that 14-day administration of pitolisant in obese mice can lead to a slight weight decrement in treated individuals. The loss was not as marked as for the reference compound, metformin (100 mg/kg bw), but still was statistically significant. The use of pitolisant for 14 days in obese mice also significantly improved glucose tolerance. It can be assumed that increased glucose tolerance may be directly related to weight loss. However, our research was of a preliminary character and did not indicated the mechanism of this action. It might be possible that this compound affects the secretion of insulin. As it was shown earlier by Nakamura et al. (2014), histamine H_3_ receptors are expressed in mouse β cells and could play a role in insulin secretion and, possibly, β cell proliferation. H_3_ histamine receptor inverse agonist JNJ-5207852 induces insulin secretion from pancreatic β cells and corrects glucose level.

Animals with induced obesity (administrated with 1% Tween 80) drank more sucrose solution than animals treated with metformin or pitolisant; however, the difference for pitolisant was not significant. Sucrose intake was associated with improved glucose tolerance for pitolisant as well as insulin sensitivity in case of metformin treatment. However, our study did not show any significant improvement in insulin resistance in obese individuals after the administration of pitolisant.

Obesity caused by high-fat diet coexists with metabolic disorders such as increased glucose, triglycerides and cholesterol levels (Dudek et al. 2015a, b; Kotańska et al. 2017). In our study, obese mice treated with pitolisant had significantly lower triglyceride levels and slightly (however not significantly) lower plasma levels of cholesterol in comparison to those in untreated animals. We suggest that improvement of these metabolic parameters may also be related to the weight loss that occurred after pitolisant treatment, but might be as well linked to another mechanism. As has been shown by Dudek et al. (2016), pitolisant reduced triglyceride levels in mice with olanzapine-induced disorders. However, obesity was not present in these studies, therefore improvement of metabolic parameters in triglyceride levels might not be associated with weight reduction.

On the other hand, one of the limitations of this study is that pitolisant was administrated only for 14 days. In fact, longer administration could show whether the weight reduction is increasing and becoming important enough to take into account as an efficient drug for the treatment of obesity. Our findings are in harmony with the results obtained with a series of H_3_ receptor inverse agonists on animals (listed by Provensi et al. 2016), but it has also been reported that histamine H_3_ receptor activation yields an anti-obese effect (Yoshimoto et al. 2006). It is also of interest that pitolisant possesses an agonistic affect at sigma-1 receptor (EMA 2015) in addition to its inverse agonistic effect at H_3_ receptors. The sigma-1 receptor is a target for obesity and eating disorders (Moore et al. 2017) and might contribute to the overall effect of pitolisant. However, one has to consider that the inverse agonistic potency of pitolisant at H_3_ receptors (EC_50_ (human receptor) of 1.5 nM) is much lower than its agonistic potency at sigma-1 receptors (EC_50_ (human receptor) of 402 nM).

With respect to the use of pitolisant in narcolepsy, the European Medicines Agency (EMA) states that, based on the available clinical data, there is no significant effect on body weight. Weight gain has been seen in 2.9% of the patients treated with pitolisant, as opposed to 1.3 and 0% of the patients treated with placebo or modafinil (the standard drug for narcolepsy treatment), respectively. However, pitolisant led to weight loss in very few patients. In some patients, effects on appetite have also been reported, which were, however, not always associated with weight gain (and/or vice versa). It is assumed that pitolisant has no direct effect on weight and that the changes in weight might have happened due to other treatment-associated factors (EMA 2015). Uguen et al. (2013) showed that in rats without metabolic disorders and obesity, pitolisant did not modify body weight during an 11-day treatment period with 10 mg/kg ip. However, none of the studies was related to obese individuals and therefore does not reflect the influence of pitolisant on body weight and metabolic parameters under this clinical condition (EMA 2015).

In conclusion, we showed that pitolisant favourably affects body weight and improves glucose tolerance and the lipid profile in obese mice. However, the level of insulin concentration after this treatment has not been determined and only such studies would allow to partially answer the question of the mechanism of glucose tolerance increment. From the screening research point of view the test groups were sufficient, but still small. Testing on larger animals groups would confirm the information obtained. Therefore, further studies are needed to elucidate the mechanism of pitolisant action in this regard.

In the present study, we showed that pitolisant may favourably affect body weight, improve glucose tolerance and correct lipid profile in obese individuals. Although the results do not indicate a high efficiency of weight loss in the mouse obesity model, it might still be considered valuable. Conducted studies have shown that pitolisant not only does not disturb glucose tolerance, but it can even improve insulin resistance in obese animals. This therefore indicates its safety in obese individuals.
